# Isotopic Incorporation Rates and Discrimination Factors in Mantis Shrimp Crustaceans

**DOI:** 10.1371/journal.pone.0122334

**Published:** 2015-04-02

**Authors:** Maya S. deVries, Carlos Martínez del Rio, Tate S. Tunstall, Todd E. Dawson

**Affiliations:** 1 Department of Integrative Biology, University of California, Berkeley, California, United States of America; 2 Department of Zoology and Physiology, University of Wyoming, Laramie, Wyoming, United States of America; Union College, UNITED STATES

## Abstract

Stable isotope analysis has provided insights into the trophic ecology of a wide diversity of animals. Knowledge about isotopic incorporation rates and isotopic discrimination between the consumer and its diet for different tissue types is essential for interpreting stable isotope data, but these parameters remain understudied in many animal taxa and particularly in aquatic invertebrates. We performed a 292-day diet shift experiment on 92 individuals of the predatory mantis shrimp, *Neogonodactylus bredini*, to quantify carbon and nitrogen incorporation rates and isotope discrimination factors in muscle and hemolymph tissues. Average isotopic discrimination factors between mantis shrimp muscle and the new diet were 3.0 ± 0.6 ‰ and 0.9 ± 0.3 ‰ for carbon and nitrogen, respectively, which is contrary to what is seen in many other animals (e.g. C and N discrimination is generally 0–1 ‰ and 3–4 ‰, respectively). Surprisingly, the average residence time of nitrogen in hemolymph (28.9 ± 8.3 days) was over 8 times longer than that of carbon (3.4 ± 1.4 days). In muscle, the average residence times of carbon and nitrogen were of the same magnitude (89.3 ± 44.4 and 72.8 ± 18.8 days, respectively). We compared the mantis shrimps’ incorporation rates, along with rates from four other invertebrate taxa from the literature, to those predicted by an allometric equation relating carbon incorporation rate to body mass that was developed for teleost fishes and sharks. The rate of carbon incorporation into muscle was consistent with rates predicted by this equation. Our findings provide new insight into isotopic discrimination factors and incorporation rates in invertebrates with the former showing a different trend than what is commonly observed in other animals.

## Introduction

Mantis shrimp (Stomatopoda) are marine crustaceans with highly specialized feeding morphology that produces among the fastest and most powerful strikes in the animal kingdom [1,2]. Although these raptorial appendages are thought to have evolved for crushing hard-shelled prey [3,4], there are anecdotal observations of mantis shrimp capturing evasive, soft-bodied prey as well [4,5], suggesting that these animals are not limited to a diet of hard-shelled prey. Understanding mantis shrimp trophic ecology and its relation to the evolution of the predatory strike depends on accurate reconstructions of mantis shrimp diets. Yet, it has been challenging to study their diets, because mantis shrimp are difficult to observe in the wild and they digest their prey too rapidly for accurate identification of stomach contents [3,4,6].

Stable carbon and nitrogen isotope ratios (i.e., ^13^C/^12^C and ^15^N/^14^N, expressed as δ^13^C and δ^15^N, respectively, and reported in parts per thousand as ‰) can provide novel insights into the trophic ecology of animals that other methods cannot [7]. Isotopic compositions of different prey items have been shown to record a predator’s diet with reliable fidelity when prey are consumed, metabolized, and assimilated into a consumer’s tissues (reviewed in [8]). Another important benefit of stable isotope analysis is that, unlike traditional methods that only document a snap-shot of a predator’s diet, stable isotope analysis can reconstruct diet assimilated over various time windows [9–11]. The time window that a tissue represents is determined with diet shift experiments designed to measure the rates of incorporation, or turnover, and the resident times of different elements in a tissue [11]. Specifically, individuals are provided with a new diet that is isotopically distinct from the former diet, and the time required for the consumer’s tissue to assimilate and reach a steady state with this new diet is measured. Different tissues incorporate the new diet at different rates. Muscle tissue, for example, exhibits slower incorporation rates than blood plasma. Recent research in birds, fish, and sharks has also shown that incorporation rates are negatively correlated with body size, whereby larger animals have slower incorporation rates than smaller animals [12–14]. Incorporation rates are governed by both tissue growth and the catabolic processes that result in tissue replacement (reviewed in [7]). The contribution of growth versus replacement of catabolized tissue to incorporation rates varies among species and even within individuals depending on physiological state [11,15–19]. Growth is the dominant determinant of isotopic turnover in rapidly growing organisms, whereas replacement of catabolized tissue represents the main contribution to turnover in non-growing animals [20,21].

Another important parameter that can be measured from diet shift experiments is isotopic discrimination, which is the difference between the stable isotope ratios of the consumer and the food resource [22]. Estimating the proportional contributions of different prey items to the diet with diet reconstruction models relies on accurate measures of discrimination [23]. ^15^N and ^13^C isotopic discrimination factors have traditionally been thought to be 3 ‰ and 0–1 ‰, respectively [24,25], but these values have been shown to vary between 0–8 ‰ depending on diet and tissue types [26,27]. Thus, empirically determined discrimination factors are the most reliable for use in diet reconstruction models [7].

While a considerable body of literature exists on discrimination factors in invertebrates (reviewed in [28]), most studies on isotopic incorporation have been conducted in birds, mammals, and fish (reviewed in [7]). The few studies on invertebrates were conducted on whole body tissue. To our knowledge, only one study on lobsters measured isotopic incorporation in more than one crustacean tissue [19]. These results showed that the rates of both hemolymph and muscle were over 100 days, which counters findings of different tissues having markedly different rates in vertebrates. Our goal, therefore, was to measure the carbon and nitrogen incorporation rates and discrimination of muscle and hemolymph in the mantis shrimp species, *Neogonodactylus bredini*, to determine whether multiple tissue types could be used to reconstruct diet over various timeframes. We expected the stable isotope incorporation rates of muscle to be slower than those of hemolymph and the discrimination factors to be similar to the average literature values of 3 ‰ and 0 to 1 ‰ for nitrogen and carbon, respectively (reviewed in [28]). To determine how mantis shrimp incorporation rates relate to those measured in other ectotherms, we compared the rates determined here to those predicted by an allometric equation derived for fish and expanded for sharks [13,29].

Given that sample preparation methods for marine invertebrates differ widely [30], a secondary goal was to test treatment methods for mantis shrimp tissues. Specifically, we compared the stable isotope ratios of delipidified and untreated tissues, because lipid δ^13^C values are more negative relative to the proteins used in metabolism. Although carbonate minerals are often also removed from some animal tissues, we did not perform these procedures, because crustacean soft tissues are known to contain low levels of carbonate minerals [30].

## Methods

### Animal acquisition and care

Autoridad Nacional del Medio Ambiente (Permit No. SEX/A-133-08) issued the collecting permit to collect animals at Galeta Marine Reserve, Smithsonian Tropical Research Institute, Colon, Panama. One-hundred *Neogonodactylus bredini* (Crustacea: Stomatopoda: Gonodactylidae; [31]) were captured from 06-Dec-2008 to 12-Dec-2008. Animals were transported to the Department of Integrative Biology at the University of California at Berkeley, where they were maintained in individual plastic cups with 0.2 L of 34–36 parts per thousand artificial saltwater at 25°C. Water temperature was kept constant by maintaining the aquarium room at 25°C. Although housing animals in this volume of water is standard protocol for mantis shrimp of this size [32], we monitored the pH of the water post hoc for five feeding cycles to determine if animal respiration or particulate organic matter in the cups altered water chemistry. We found that, on average, the pH of the cups was reduced to 0.2 below ambient.

### Diet shift Experiment

For 45 days, from the time of capture until the start of the diet-switch experiment, animals were fed the Western Atlantic, intertidal snail, *Cerithium eburneum*. This relatively short timeframe was thought to be sufficient for individuals to assimilate the stable isotope composition of *C*. *eburneum*, based on a one month timeframe used in an incorporation rate study in rock lobsters [19]. After the 45 days, individuals were switched to a diet of the Pacific, temperate, rocky intertidal snail, *Tegula funebralis*, which had stable isotope values that were different from *C*. *eburneum* (Table 1) and the soft tissue was approximately 15% of the mantis shrimps’ body mass. Animals received whole snails every three days for the duration of the experiment. After feeding, the mantis shrimp had leftover snail tissue in their cups. We, therefore, considered the feeding schedule to be *ad-libitum*. After 6 hours, the water in the cups was changed to prevent contamination from decaying snail material and fecal matter.

**Table 1:** Means ± standard deviation (s.d.) of δ^13^C and δ^15^N for *N*. *bredini*’s tissues before and after reaching an asymptotic value.

	Mean δ^13^C ± s.d. (‰)		Mean δ^15^N ± s.d. (‰)	
Tissue type	Day 0	Day 292	Day 0	Day 292
Muscle	-9.9 ± 1.7	-11.6 ± 0.3**	7.9 ± 0.6	11.2 ± 0.3**
	n = 9	n = 3	n = 9	n = 3
Hemolymph	-10.6 ± 0.6	-13.8 ± 0.3*	6.4 ± 0.3	10.0 ± 0.6***
	n = 9	n = 3	n = 9	n = 3
*C*. *eburneum*	-8.7 ± 0.8	NA	4.6 ± 0.3	NA
(initial diet)	n = 20		n = 20	
*T*. *funebralis*	NA	-14.9 ± 0.6	NA	9.9 ± 0.3
(final diet)		n = 15		n = 10

Mean δ-values for the initial diet of *C*. *eburneum* and the final diet of *T*. *funebralis* in the “Day 0” and “Day 292” columns, respectively. n = sample size,

* = P<0.05,

** = P<0.01,

***P<0.001 represent significant differences between the Day 0 and Day 292 stable isotope values, as calculated from Welch’s two-sampled t-tests.

To establish initial growth parameters, total body length (measured from the distal tip of the rostrum to the posterior end of the telson), carapace length, and the weights of all individuals were measured one day prior to switching *N*. *bredini* to the new diet. Total body lengths ranged from 33–49 mm and all individuals were sexually mature adults (adult minimum size = 30 mm; [33]). Lengths were measured with digital calipers to the nearest mm (Absolute Coolant Proof Caliper Series 500, Mitutoyo Corporation, Takatsu, Japan). Wet weight was measured three times at 15-second intervals to the nearest μg.

On Day 0 of the experiment, nine individuals were randomly selected to be measured, weighed, euthanized on ice, and dissected. The δ^13^C and δ^15^N values from these individuals were used to establish initial stable isotope values of both hemolymph and muscle. All other animals were switched to the new diet of *T*. *funebralis*. Throughout the experiment, 3 to 6 individuals were randomly sampled 1, 2, 4, 7, 13, 19, 25, 31, 40, 64, 97, 127, 160, 193, 223, or 292 days after being switched to the new diet. Molts were recorded for the duration of the experiment. Eight individuals (9%) died during the experiment and were not included in analyses.

### Sample collection and preparation

Once euthanized, approximately 0.2 ml of hemolymph was extracted with a 27-gauge needle and a 1 ml syringe by inserting the needle above the fifth abdominal somite. Muscle from abdominal somites 2–6 was also dissected. Tissues were stored frozen at -20°C until preparation for stable isotope analysis. Ninety-two muscle and 63 hemolymph samples from *N*. *bredini* were freeze-dried (FreezeZone 12 Liter Freeze Dry System, Labconco, Kansas City, MO, USA) for 48 hours. The number of hemolymph samples is smaller than the number of muscle samples, because in some cases the hemolymph samples were too small for analysis. To acquire the target sample size of hemolymph, vials containing the samples were placed in coin envelopes and frozen at -80°C for 12 hours before being freeze-dried. Once dry, muscle and hemolymph samples were homogenized. Twenty *C*. *eburneum* and ten *T*. *funebralis* muscle samples were also freeze-dried and homogenized.

Lipids were extracted from 44 *N*. *bredini* muscle samples and 10 *T*. *funebralis* with petroleum ether, following recommendations for crustacean muscle in [30] and [34]. Each homogenized muscle sample was first separated into two aliquots. One aliquot was immediately prepared for stable isotope analysis (see below), while the other was treated by immersing it in 0.2 ml of petroleum ether. Treated samples were agitated for 30 sec and then left undisturbed for 30 min twice. The supernatant was then discarded. To remove any leftover solvent, treated samples were re-frozen at -20°C for 12 hours and then placed in the freeze-drier for twelve hours.

### Stable isotope analysis

All samples were placed in tin capsules (5 x 9 mm, Costech Analytical Technologies, Valencia, CA, USA) and weighed to 180 ± 50 μg for muscle and 220 ± 50 μg for hemolymph. δ^13^C and δ^15^N were analyzed with elemental analyzer/continuous flow isotope ratio mass spectrometry at the Center for Stable Isotope Biogeochemistry at UC Berkeley using a CHNOS Elemental Analyzer (vario ISOTOPE cube, Elementar, Hanau, Germany) coupled with an IsoPrime100 Isotope Ratio Mass Spectrometer (Isoprime, Cheadle, UK). Isotope ratios are expressed using δ notation as:

$$\delta^{h}X=\left(\frac{R_{sample}}{R_{standard}}-1\right)\times \;1000$$ 1

where *X* is the element, *h* is the high mass number, *R* is the high mass-to-low mass isotope ratio, and *R*_*standard*_ is Vienna Pee Dee belemnite (VPDB) for carbon and AIR for nitrogen. Units are parts per thousand (per mil, ‰). We used peach leaves (Standard Reference Material [SRM] No. 1547, n = 105, mean ± standard deviation: δ^13^C = -25.9 ± 0.1 ‰ and δ^15^N = 2.1 ± 0.1 ‰) and bovine liver (SRM No. 1577, n = 38, δ^13^C = -21.54 ± 0.1 ‰ and δ^15^N = 7.9 ± 0.1 ‰) as references to correct the stable isotope ratios for drift and linearity of the mass spectrometer.

### Statistical analysis

All statistical analyses were performed using either the JMP (v. 9.0, SAS Institute Inc., Cary, NC, USA) or R (package: nlme, v. 2.15.0, R Development Core Team, The R Foundation for Statistical Computing, Vienna, Austria) statistical packages. To determine whether lipid extraction had the expected effect of yielding δ^13^C values that were more enriched than those of the untreated tissue, paired *t*-tests were used to compare the stable isotope ratios of the lipid-extracted aliquots to the ratios of the untreated aliquots. The carbon to nitrogen molar ratio (C:N) was also calculated for all tissues. Because the C:N ratio closely correlates with tissue lipid content, it can be used to estimate the lipid content in a tissue [35].

The time course of isotopic incorporation after the diet shift was modeled with a one-compartment, first-order kinetics model from [36]:

$$\delta^{h}X_{t}=\;\delta^{h}X_{\infty }-\left(\delta^{h}X_{\infty }-\delta^{h}X_{0}\right)e^{\left(-\frac{t}{\tau }\right)}$$ 2

where δ^*h*^ *X*_*t*_ is the stable isotope value at time (*t*), δ^*h*^ *X*_*∞*_ is the estimate of the final stable isotope value when the predator tissue has reached a steady state with the new diet, δ^*h*^ *X*_*0*_ is the initial isotopic value, and τ is the average residence time or the time to reach a steady state with the new diet. We chose a one-compartment incorporation model after first analyzing the data with the reaction progress model [37], which determined that both muscle and hemolymph exhibited one compartment kinetics.

Many studies report average residence time (τ) as the fractional turnover rate or λ, where λ = 1/τ. We report both λ and τ, but the model was calculated with τ because this parameter provides an intuitive measure of residence time in days [36,38]. From τ, the isotopic half-life was calculated as ln(2)τ (or ln(2)/λ) for both muscle and hemolymph.

To determine whether differences between individuals, including the sex of the individual and whether it molted during the experiment, influenced muscle incorporation rates, we modified a random effects model created by [39] for the muscle stable isotope data. Model fits were compared with the Akaike information criterion (AIC).

The diet-to-tissue discrimination factor also can be calculated from the incorporation rate model. Discrimination factors were calculated for both isotopes as:

$$\Delta^{h}=\delta^{h}X_{\infty \;mantis\;shrimp}-\delta^{h}X_{prey}$$ 3

where Δ^*h*^ is the discrimination factor for *h* stable isotope, δ^*h*^ *X*_*∞ mantis shrimp*_ is the estimated value of the steady-state isotopic composition of the mantis shrimp tissue and δ^*h*^ *X*_*prey*_ is the mean isotopic value of new diet (*T*. *funebralis*).

To measure growth over the course of the experiment, the mass or carapace length of each individual on Day 0 was subtracted from the respective final measurement at the time that the individual was euthanized. Significant changes in these growth variables were evaluated using least-squares linear regression models. A common practice in incorporation rate studies is to calculate the percent contributions of growth versus tissue replacement via catabolism to incorporation rates following [40]. However, we were unable to apply this model to our dataset, because the growth rates were either zero or negative and the model requires positive growth.

## Results

### Delipidification treatment

The mean ± standard error (s.e.) C:N ratio of *N*. *bredini* muscle was 3.5 ± 0.3 and hemolymph was 3.5 ± 0.4. The C:N ratio for *T*. *funebralis* whole tissue was 3.9 ± 0.3. We found a small but significant difference between the lipid extracted and untreated treated muscle aliquots for δ^15^N in *T*. *funebralis* (mean difference [MD] ± s.e. = 0.2 ± 0.1 ‰, paired *t*-test: *P* = 0.01, *t*_9_ = 3.04, n = 10). However, there were no significant differences between the isotopic compositions of the treated and untreated muscle tissues in *N*. *bredini* (δ^13^C: MD ± s.e. = 0.1 ± 0.3 ‰, paired *t*-test: *p* = 0.30, *t*_43_ = 0.49, n = 44; δ^15^N: MD ± s.e. = 0.1 ± 0.3 ‰, paired *t*-test: *p* = 0.75, *t*_42_ = 0.32, n = 43) and for δ^13^C in *T*. *funebralis* (MD ± s.e. = 0.0 ± 0.1 ‰, paired *t*-test: *p* = 0.70, *t*_9_ = -0.40, n = 10). We therefore decided to exclude the lipid extracted δ^13^C values from incorporation rate and discrimination calculations.

### Discrimination (Δ)

**Fig 1:**
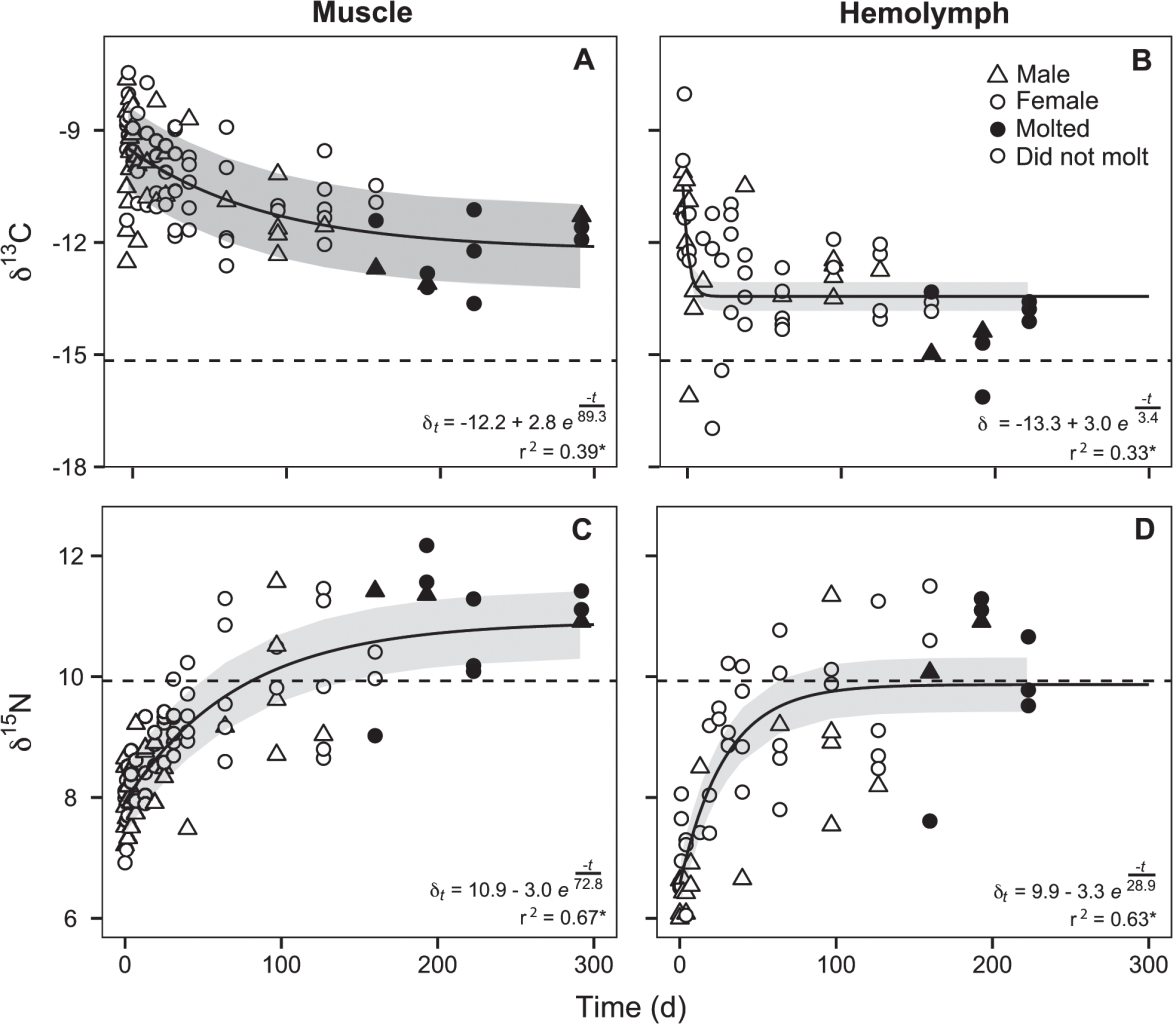
*N*. *bredini* stable isotope ratios as a function of time after a diet shift. One-compartment models (solid lines) sufficiently described the changes in δ^13^C (A, B) and δ^15^N (C, D) for muscle (A, C) and hemolymph (B, D) as a function of time after a diet shift. To illustrate variation between experimental *N*. *bredini*, males (triangles) and females (circles) that molted at least once during the study (black shapes) or did not molt (open shapes) are coded. Dashed lines are mean stable isotope ratios of the new diet.

The final *T*. *funebralis* diet had mean δ^13^C and δ^15^N values that were significantly different from those of the initial *C*. *eburneum* diet (Table 1; Welch’s two sample t-test: δ^13^C, t = 25.54, d.f. = 27.16, P<0.0001, δ^15^N, t = -53.33, d.f. = 26.44, P<0.0001). Isotopic discrimination (Δ) factors for *N*. *bredini* were much greater for δ^13^C than for δ^15^N (Table 2). This pattern was especially pronounced for δ^13^C in muscle, as none of the predators’ final isotope ratios overlapped with the mean value of the new diet (Fig 1). In contrast, δ^15^N in muscle and hemolymph exhibited small Δ values (Table 2).

**Table 2:** Model parameters ± standard error from one-compartment isotopic incorporation rate models.

Tissue	Isotope	n	Residence time τ (d)	Equilibrium value δ_∞_ (‰)	Initial-final value δ _0_ - δ_∞_ (‰)	*r* ^2^ for model fit	Fractional turnover rate λ (d^-1^)	Half-life (d)	Discrimination Δ (‰)
M	^13^C	92	89.3 ± 44.4	-12.2 ± 0.6	-2.8 ± 0.5	0.39*	0.011 ± 0.006	61.9 ± 30.8	3.0 ± 0.6
M	^15^N	92	72.8 ± 18.8	10.9 ± 0.3	3.0 ± 0.30	0.67*	0.014 ± 0.004	50.4 ± 13.0	0.9 ± 0.3
H	^13^C	61	3.4 ± 1.4	-13.3 ± 0.2	-3.0 ± 0.6	0.33*	0.293 ± 0.150	2.4 ± 1.2	1.7 ± 0.3
H	^15^N	63	28.9 ± 8.3	9.9 ± 0.2	3.3 ± 0.3	0.63*	0.035 ± 0.010	20.0 ± 5.7	0.1 ± 0.2

Fractional turnover rates calculated from λ=1/|τ, half lives, and discrimination values are also presented. M = muscle tissue, H = hemolymph tissue, n = sample size.

* = *P* < 0.001.

### Tissue incorporation rates

A one-compartment non-linear model for incorporation fit to the untreated stable isotope ratios estimated incorporation rates and asymptotic δ-values (Table 2 and Fig 1). As predicted, the isotopic incorporation rate of hemolymph was faster than that of muscle, but the final asymptotic values were similar (Table 2 and Fig 1). The average residence time ± standard error of ^13^C in hemolymph was 3.4 ± 1.4 days compared to a residence time of 28.9 ± 8.3 days for ^15^N (Table 2). Within muscle, the average residence times of ^13^C and ^15^N were similar (Table 2). After 200 days, the isotopic values of muscle were within the 95% confidence intervals for the estimated asymptotic values (δ_∞_), suggesting that the tissue had established a steady-state isotopic composition with the new diet (Fig 1). The mean values of δ^13^C and δ^15^N for hemolymph were within the 95% confidence intervals of the asymptotic values estimated by models (Fig 1). Given that the incorporation rates of hemolymph were so rapid, we assume that the estimates of the asymptotic values are correct. Including sex of the individuals and whether or not they molted during the experiment in the random effects models of incorporation rate did not change the AIC scores for either stable isotope in muscle, likely because of the small samples sizes of males and of individuals that molted during the study (Fig 1).

### Growth

**Fig 2:**
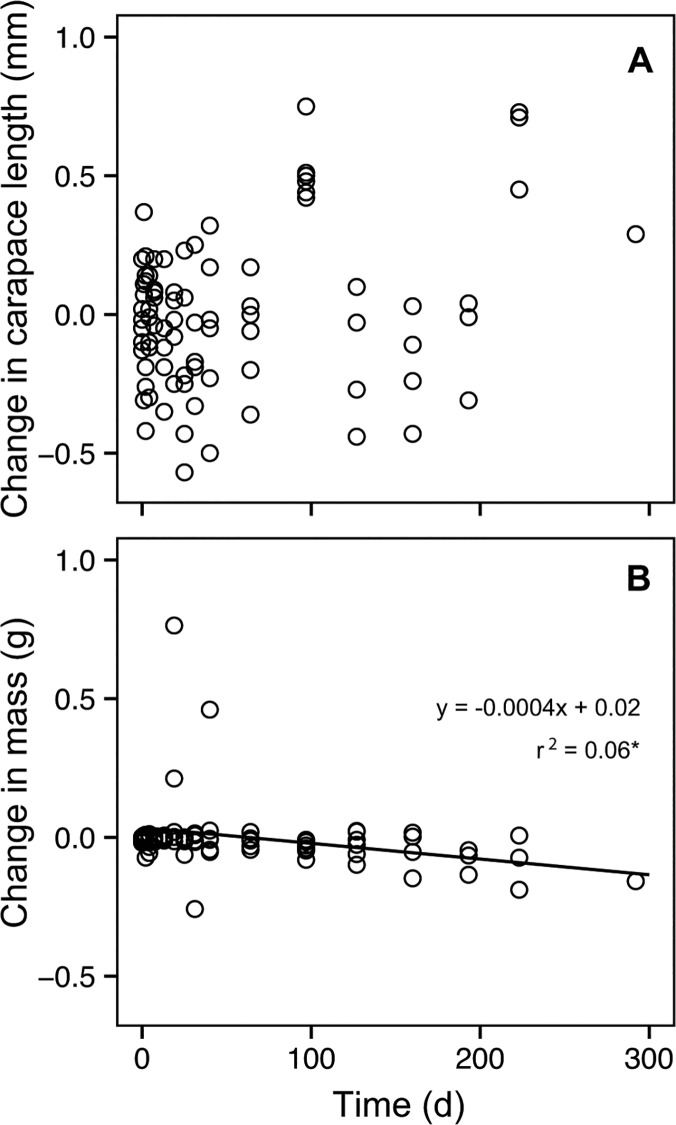
Growth as a function of time after a diet shift. Growth for the duration of the study was examined by measuring the change in carapace length (A) and the change in mass (B) from the start of the experiment to the time of tissue collection for each individual (open circles). There was a significant change in mass (regression line represented by solid line in B) but not in carapace length.

The change in carapace length from the start of the experiment to the time of tissue collection was not significantly different from zero (least-squares linear regression: *r* ^2^ = 0.03, *P* = 0.06, *F*_1,87_ = 3.78, n = 89; Fig 2). The change in body mass from the start of the experiment to the time of tissue collection decreased slightly over time (least-squares linear regression: slope = -0.0004 g/days, *r* ^2^ = 0.06, *P* = 0.01, *F*_1,87_ = 6.72, n = 89; Fig 2).

## Discussion

Patterns of incorporation and discrimination in *N*. *bredini* were consistent with those observed in both invertebrate and vertebrate taxa. However, a few of our results were counter to our initial predictions. First, delipifying samples did not significantly enrich δ^13^C values. Second, the discrimination factors for both tissues were not in the commonly cited 0–2 ‰ range for Δ^13^C nor around 3 ‰ for Δ^15^N. Third, the animals did not grow during the experiment, suggesting that all components of incorporation can be attributed to maintenance metabolism. Finally, the incorporation rate of ^15^N in hemolymph was considerably slower than that of ^13^C. Here, we discuss these results, and conclude by showing that, despite the unexpected results, the rate of carbon incorporation into muscle was consistent with rates predicted by an allometric equation developed for vertebrate aquatic ectotherms.

### Delipidification treatment

When the lipid concentration of a tissue is consistently low, removing lipids does not necessarily alter δ^13^C values [35]. The C:N ratio of both *N*. *bredini* tissues was ≈ 3.5, which corresponds to a lipid content of less than 5% [35] and likely explains why lipid extraction did not yield significantly higher δ^13^C values. The results were similar for the δ^13^C values in *T*. *funebralis*, even though the C:N ratio of the tissue was 3.9. However, the δ^15^N values from delipified *T*. *funebralis* muscle were significantly higher than those of untreated samples, suggesting that the petroleum ether solvent extracted a fraction of tissue possibly containing lipoproteins [34]. Based on these findings, we used the stable isotope values from the untreated tissues in all analyses. We further presume that there are no strong reasons to extract lipids from mantis shrimp or *T*. *funebralis* tissues in future stable isotope research on these taxa.

### Discrimination factors of C and N isotopes

Although the Δ^13^C of 2–4 ‰ and Δ^15^N of 0–1 ‰ measured here deviate from commonly cited Δ-values in the literature (0 to 1 ‰ for Δ^13^C and ~3 ‰ for Δ^15^N; reviewed in [26]), they are actually similar to those found in other animals, including true shrimp, pigs, locusts, and leopard sharks for Δ^13^C (range 1.7–3.5 ‰; reviewed in [14,26,41]), and true shrimp, blue crabs, red drum fish, and quail for Δ^15^N (range: 0.9–1.0 ‰; reviewed in [26,41]). The wide range of discrimination values exhibited by these animals has been attributed to variation in the nutritional value of different experimental diets.

The high protein quality and quantity of the experimental diet likely contributed to *N*. *bredini*’s low Δ^15^N values. While our experiment provided no information on the effects of food quality on Δ values, other animals, including mammals, birds [42], and brittle stars [43] have exhibited significant decreases in Δ^15^N in response to being fed high quality protein diets (defined by a high content of the most limiting essential amino acids). *T*. *funebralis* likely provided a high protein quality diet because it has high concentrations of essential (and often limiting) amino acids across its tissue types [44]. In crustaceans, low Δ^15^N values can also result from consuming diets with a high protein content [41,45,46], which have C:N ratios less than 6 [47]. *T*. *funebralis’* C:N ratio is 3.9, suggesting that its muscle, and likely also its other tissues [44], are high in protein content. We therefore hypothesize that the low Δ^15^N values found in mantis shrimp are a consequence of the high protein content and quality of their experimental diet and the efficient use of such food.

The high Δ^13^C values measured here are similar to Δ-values measured in other crustaceans, including Pacific white shrimp [41], ghost shrimp [47], oysters [48], and prawns [49]. For invertebrate tissues, high Δ^13^C values are often attributed to high concentrations of calcium carbonate [30]. Yet, most crustacean tissues, including the exoskeleton, have low carbonate contents [30,47]. The low C:N ratio measured in *N*. *bredini* further indicate that carbonates were not present in appreciable amounts in muscle and hemolymph tissues [50]. Thus, the high Δ^13^C values measured here and in other crustaceans cannot be fully explained by elevated amounts of calcium carbonate in tissues. These findings point to the possibility of a general trend for considerably high Δ^13^C in crustaceans and highlight the need for experimentation that determines the underlying mechanism for this observation [47].

Alternatively, due to the low sample size on the final sampling day, we cannot rule out the possibility that the carbon isotopic compositions of hemolymph and muscle did not achieve an asymptotic value with the new diet. However, the final δ^13^C values of muscle fall within the 95% confidence interval of the asymptote estimated by the incorporation model (Fig 1). While not all of the final δ^13^C values of hemolymph fall within the 95% confidence interval, the rapid incorporation rate of ^13^C in hemolymph strongly suggests that the estimates of the ^13^C asymptotic value, and therefore the discrimination factor, are correct.

### Incorporation rates of C and N isotopes

In contrast to findings in lobsters that both hemolymph and muscle have incorporation rates of over 100 days [19], we found markedly different incorporation rates between and within these two tissues. The incorporation rate of *N*. *bredini* hemolymph was especially intriguing. Unlike in muscle, the incorporation rates of hemolymph nitrogen and carbon differed dramatically. The average residence time was 29 days for nitrogen but only 3 days for carbon (Table 2). As expected from a tissue that integrates a variety of dietary inputs over very short time scales, the initial isotopic values in hemolymph were variable both at the beginning and throughout the experiment. Consequently, the hemolymph incorporation rates should be interpreted cautiously. However, again, given the rapid incorporation rates measured in hemolymph, we are confident in both the ^13^C and ^15^N asymptotic values. The discrepancy in turnover between carbon and nitrogen can be interpreted as faster rates of catabolizing carbon skeletons in amino acids, but the retention and recycling of amino groups [51]. Nitrogen conservation, however, is not expected in a carnivorous animal with a high protein intake like *N*. *bredini* [52]. We thus find that the hemolymph incorporation rates are perplexing and require further experimentation.

Although gender and molting did not seem to affect incorporation rates, the samples sizes of males and of the individuals that molted during the experiment were possibly too small to detect an effect. It is especially difficult to decipher the influence of molting on incorporation rates, because all of the individuals that molted were at the end of the experiment. Thus, if we had excluded molted individuals from the analysis, we would have underestimated both incorporation and discrimination. The influences of gender and molting on incorporation rates in crustaceans would benefit greatly from future study, as gender is known to impact incorporation rates in vertebrates [39,53] and molting affects protein synthesis and degradation (reviewed in [54]).

### Maintenance metabolism and reduced growth in *N*. *bredini*

Given that the experimental animals showed no significant growth throughout the experiment (Fig 2), we inferred that the changes to isotopic composition in *N*. *bredini* are attributed exclusively to catabolic processes. This observation has also been documented in determinate growers, such as mammals and birds [9,55–57]. Adult *N*. *bredini* that are at least 35 mm in length have an average intermolt growth rate of 6% and molt every 3–4 months [58]. Most individuals molted once or not at all during this study, but six individuals molted twice with intermolt periods ranging between 4–6 months. These long intermolt periods could explain why *N*. *bredini*’s growth rates and patterns of isotopic incorporation reflected those of determinate growers.

It is also possible that the animals experienced starvation during the experiment, which resulted in the slight reduction in average body mass. However, low Δ^15^N values, generally indicate that dietary nitrogen is near optimal growth requirements [26]. Additionally, we commonly found evidence of broken shells and marks on *T*. *funebralis* muscle where the tissue had been consumed. Leftover soft tissue was found in most shells, which implies that the individuals were supplied with ample food to reach satiation. We instead attribute the reduction in body mass to a common observation that mantis shrimp exhibit reduced growth in the laboratory [59].

While unlikely, laboratory conditions, including reduced temperature and altered water chemistry, could have also created stressful conditions and led to reduced growth rates. At the collection site, the range of yearly temperatures is 23–36°C, and the laboratory temperature of 25°C is common for at least three months of year (data from the Smithsonian Tropical Research Institute’s Physical Monitoring Program). Little is known about how water chemistry affects incorporation rates, but we can hypothesize that the rates measured here might have been slower if the water chemistry had remained constant.

### Body size in relation to isotopic incorporation

**Fig 3:**
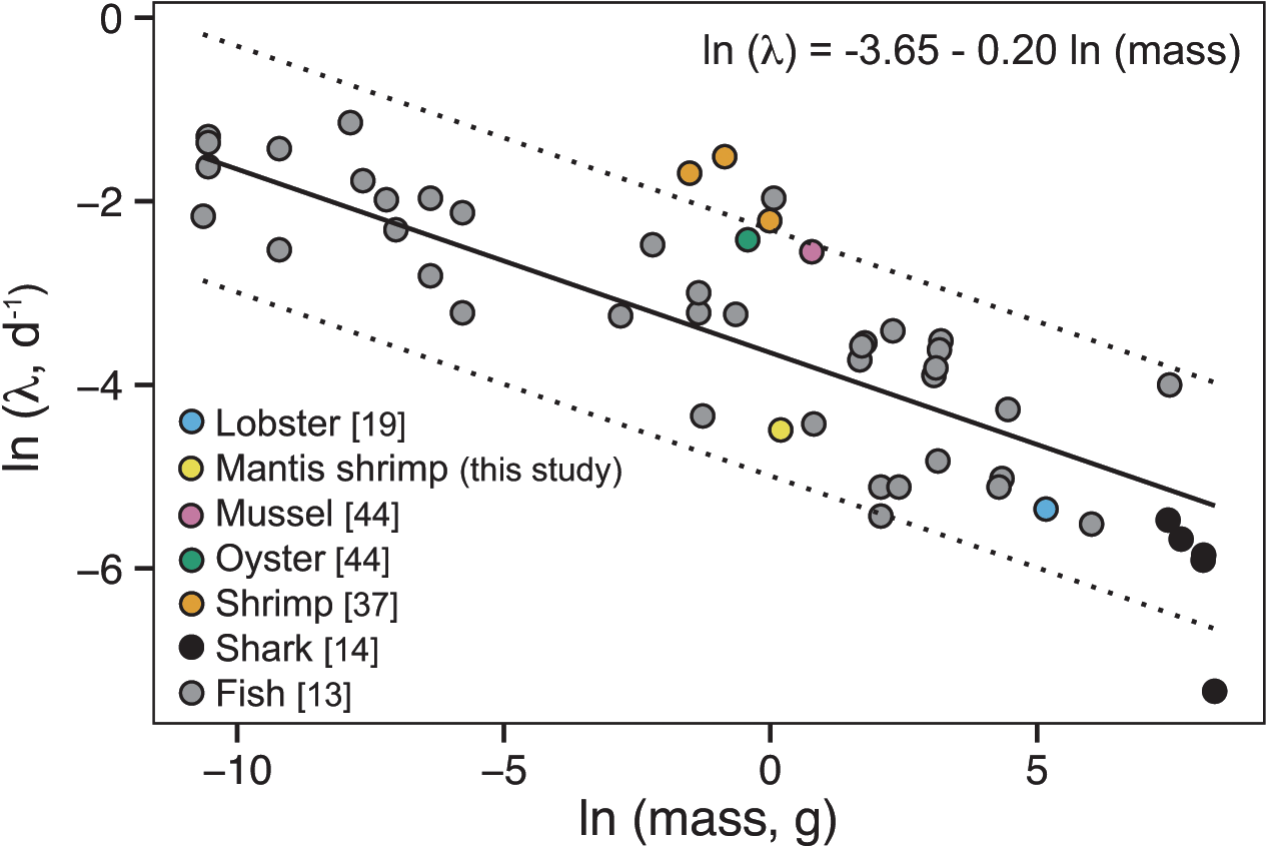
Carbon incorporation rates of *N*. *bredini*, lobsters, mussels, oysters, shrimps, sharks, and 12 teleost fish species. Carbon incorporation rates (represented as fractional turnover rate, λ) for *N*. *bredini* muscle (black circle) are within the 95% prediction intervals (dotted lines) of the allometric relationship (solid line) derived by [13] for 12 species of fishes and expanded upon by [14] for leopard sharks. Data from the whole bodies of Pacific white shrimp [37], the muscle of rock lobsters [19], and the soft tissues of blue mussels, and Pacific oysters [44] were also included in the model to further expand the analysis to crustaceans and molluscs.

Although *N*. *bredini’s* biology and life history differ from ectothermic vertebrates, its incorporation rates fall within the range of expected values based on an allometric equation relating carbon incorporation rates to body mass that was calculated for teleost fish [13] and expanded upon for sharks [14] (Fig 3). To broaden this model, we included four other invertebrates from the literature: Pacific white shrimp, blue mussels, Pacific oysters, and rock lobsters [19,41,48], for which we could calculate the fractional turnover rate (λ). Mussels, oysters, and lobsters fit within the 95% prediction intervals but Pacific white shrimp did not, possibly because the shrimp measurements were made on the whole body as opposed to only soft tissues. Despite this discrepancy, this comparison suggests the possibility of a common relationship between incorporation rate and body size across aquatic vertebrates and invertebrates. It also highlights a need to conduct more controlled laboratory experiments that measure isotopic incorporation as a function of time in aquatic invertebrates to determine the generality of this model across size ranges and taxa.

## Conclusions

Although mantis shrimp are common in many tropical and sub-tropical marine habitats, their diet has remained elusive. The empirically determined isotopic incorporation rates of *N*. *bredini’s* hemolymph and muscle will allow for determining diet assimilated over temporal scales of days and months, respectively. The discrimination factors will be integral to estimating the contribution of different prey to the diet. This information will be critical for determining the trophic ecology of many marine ecosystems.

More broadly, to our knowledge, this study is among the first to examine the incorporation rates and discrimination factors of multiple crustacean tissues. Our results revealed a possible trend in crustacean tissues that Δ^13^C is around 3‰, as opposed to 0–1‰ observed in many taxa [28]. We also demonstrated that the time points of diet assimilation represented by the muscle and hemolymph incorporation rates counter findings in rock lobsters, and instead align more closely with vertebrates. Finally, we provide evidence that there might be a general relationship between incorporation rate and body size that spans many animal taxa. Future studies examining this relationship across animals would be an integral contribution of the field of stable isotope ecology.

## Supporting Information

S1 Dataset Muscle stable isotope data. The text file entitled “S1_Dataset” is a spreadsheet with the muscle stable isotope data and the growth and molting data. The columns respond to the data as follows: TIME is the day on which the animal was sacrificed. INDIVIDUAL has the individual identification numbers. Initial_Carapace_mm and Final_Carapace_mm refer to the carapace lengths of the individuals one day prior to the start of the experiment (initial) and on the day of sacrifice (final), respectively. Initial_MASS_(mg) and Final_MASS_(mg) refer to the mass of the individuals one day prior to the start of the experiment and on the day of sacrifice, respectively. CL_change refers to the change in carapace length between the initial and final carapace length values. W_change refers to the change in mass between the initial and final masses. Molt refers to whether or not the individuals molted during the experiment (n = did not molt, y = did molt during the study). Sex is the sex of the individual (f = female, m = male). δ^15^N and δ^13^C are the nitrogen and carbon stable isotope values, respectively, represented in parts per mil (‰).
(TXT)

S2 Dataset Hemolymph stable isotope data. The text file entitled “S2_Dataset” is a spreadsheet with the stable isotope values of hemolymph and the mass and molting data. The columns respond to the data as follows: TIME is the day on which the animal was sacrificed. INDIVIDUAL refers to the individual identification numbers. Initial_MASS and Final_MASS refer to the mass of the individuals one day prior to the start of the experiment and on the day of sacrifice, respectively. Molt refers to whether or not the individual molted during the experiment (n = did not molt, y = did molt during the study). Sex is the sex of the individual (f = female, m = male). δ^15^N and δ^13^C are the nitrogen and carbon stable isotope values, respectively, represented in parts per mil (‰).
(TXT)

S3 Dataset *Tegula* and *Cerithium* stable isotope values. The text file entitled “S3_Dataset” is a spreadsheet with the stable isotope values of the muscle tissue from the initial diet, *Cerithium eburneum*, and the experimental diet, *Tegula funebralis*. The column Species, refers to whether the tissue was from *C*. *eburneum* (C) or *T*. *funebralis* (T). The columns entitled δ^15^N and δ^13^C are the nitrogen and carbon stable isotope values, respectively, represented in parts per mil (‰).
(TXT)

S4 Dataset Comparison of treated tissues. The comma separated values (.csv) file entitled “S4_Dataset” is a spreadsheet with the stable isotope values of the untreated and delipidified muscle tissue from a subset of *N*. *bredini* and *T*. *funebralis* individuals. Individual refers to the individual identification number. Species refers to whether the tissue was from *N*. *bredini* (N) or *T*. *funebralis* (T). Treated_untreated refers to whether the tissues underwent delipidification procedures (treated) or was not treated (untreated). The columns entitled δ^15^N and δ^13^C are the nitrogen and carbon stable isotope values, respectively, represented in parts per mil (‰).
(CSV)
